# Illinois State Veterinary Medical Association

**Published:** 1890-04

**Authors:** J. F. Pease

**Affiliations:** Recording Secretary; Quincy, Ill.


### SOCIETY PROCEEDINGS.

*The Illinois State Veterinary Medical Association* held its regular semi-annual meeting at the National House, Peoria, Thursday, February 20th.

The following members were present: J. A. Calder, C. E. Hollingsworth, James McClintock, J. T. Nattress, J. F. Pease, H. G. Pyle, J. D. Robinson, J. F. Reid, John Scott, R. W. Story, H. A. White, W. L. Williams, H. Thompson, S. H. Kingery, and A. G. Alverson.

Order was called at 11 A. M., President Williams in the chair. After the roll-call and the reading of the minutes, the following were proposed for membership and unanimously elected: J. D. Rutherford, Ontario, '87, Rock Island; S. V. Ramsey, Chicago, '89, Tuscola; W. A. Smith, Chicago, '89, Sparland.

The treasurer's report was read and approved.

Dr. Kingery then reported quite fully on a case of coal-oil poisoning.

DISCUSSION.—Dr. Williams suggested that similar symptoms might have been produced from strangling in drenching. Dr. Nattress has seen ill-effects from the too free application of the oil to the surface, to kill lice. (?) Might this not be from interference with the functions of the skin?

Dr. Butler, president of the Iowa association, was then introduced to the members, after which the meeting adjourned.

At 2 P. M., the members came to order to listen to an interesting paper on actinomycosis by Dr. John Scott, of Peoria.

DISCUSSION.—Dr. Butler attacked the position of the author on the constitutional nature of the disease; the fungus is essentially an *invading* one; its spores are too large to be readily carried by the vascular system.

Dr. Pease quoted from Dr. Billings to prove the invading nature of the fungus. Dr. Williams thought that all the tissues attacked could be easily invaded from epithelial surfaces.

As a whole, the society coincided wlth the author on the sanitary importance of destroying the carcases of all animals at all infected with the disease.

Dr. Williams asked if there is any authenticated recovery from the disease. Dr. Butler thinks he had a genuine recovery after extirpation of the thyroid gland. The author read from the State report to prove that extensive internal lesions had been found where the external lesions were very slight.

Dr. J. F. Reid, of Decatur, presented a well-written paper on the "Use of Anæsthetics in Veterinary Practice."

The discussion brought out the point that Squibb's chloroform is the best and cheapest, especially as an occasional fatality may occur, and the surgeon be exposed to censure.

Correspondence was received from the secretary of the Indiana association, thanking this society for its invitation to attend this meeting.

Word was received from Dr. Tiffiny explaining his failure on programme.

Dr. Tait Butler then addressed the Society on the subject of the United States Veterinary Association. He presented correspondence from Dr. Michener announcing the holding of the next meeting in Chicago in September. A discussion ensued on the duty of this society toward the national society. On motion of Dr Story, seconded by Dr. Pease, a committee to consist of the president, corresponding secretary and one member to be appointed by the chair, was created to solicit new members in Illinois. The chair appointed Prof. A. H. Baker.

The Committee on Arrangements reported that they had interviewed the editor of the *Review* on the subject of printing the papers and reports of our meetings. He offers to print all the papers and furnish 100 reprints at a nominal expense. The committee advised that the offer be accepted. On motion by Dr. Scott the matter was referred to the president with power to act.

Votes of thanks were tendered to Dr. Butler for his attendance, and to the essayists.

An amendment to Article I of the by-laws was proposed in writing, signed by Drs. S. H. Kingery and H. G. Pyle, and was read by the secretary, and laid over till the November meeting.

The Association then adjourned until the annual meeting.

QUINCY, ILL. J. F. PEASE, *Recording Secretary.*

