# A Correction

**Published:** 1890-07

**Authors:** Tait Butler

**Affiliations:** Davenport, Ia.


### A CORRECTION.

*Editor of* THE JOURNAL OF COMPARATIVE MEDICINE AND VETERINARY ARCHIVES.

In reading the report of the proceedings of a late meeting of the Illinois State Veterinary Medical Association, published in the April number of your journal, I notice the following :

"At 2 P.M. the members came to order to listen to an interesting paper on 'Actinomycosis,' by Dr. John Scott, of Peoria.

"*Discussion.*—Dr. Butler attacked the position of the author on the constitutional nature of the disease ; the fungus is essentially an invading one ; its spores are too large to be readily carried by the vascular system."

It is, perhaps, of minor importance what my views are on any such subject, but I certainly object to being credited with maintaining such views in regard to the question at point. I most assuredly made no such statement, but merely asked the essayist if the "spores" had been identified and demonstrated to be small enough to be carried by the blood through the capillary system. I also asked if a preponderance of evidence justified an unqualified statement that the fungus was not "essentially an invading one?"

Being requested by the President to open the discussion, I asked those questions for the purpose of directing it along that line which I thought of most importance, and to that fact, no doubt, is the error of the reporter due. Yours respectfully,

TAIT BUTLER.

*Davenport, Ia., June 14th,* 1890.

